# Computational simulation of the predicted dosimetric impact of adjuvant yttrium-90 PET/CT-guided percutaneous ablation following radioembolization

**DOI:** 10.1186/s13550-016-0244-1

**Published:** 2016-12-12

**Authors:** Alexander S. Pasciak, Abigail Lin, Christos Georgiades, Laura K. Findeiss, Shannon Kauffman, Yong C. Bradley

**Affiliations:** 1 Department of Radiology, University of Tennessee Graduate School of Medicine, Knoxville, TN USA; 2 School of Medicine, The Johns Hopkins Hospital, 733 N Broadway, Baltimore, MD 21205 USA; 3 Department of Radiology, The Johns Hopkins Hospital, Baltimore, MD USA; 4 Department of Radiology, Miami Valley Hospital, Dayton, OH USA

**Keywords:** Yttrium-90, Y90, Radioembolization, Interventional Oncology, SIRT, Ablation

## Abstract

**Background:**

^90^Y PET/CT post-radioembolization imaging has demonstrated that the distribution of ^90^Y in a tumor can be non-uniform. Using computational modeling, we predicted the dosimetric impact of post-treatment ^90^Y PET/CT-guided percutaneous ablation of the portions of a tumor receiving the lowest absorbed dose. A cohort of fourteen patients with non-resectable liver cancer previously treated using ^90^Y radioembolization were included in this retrospective study. Each patient exhibited potentially under-treated areas of tumor following treatment based on quantitative ^90^Y PET/CT. ^90^Y PET/CT was used to guide electrode placement for simulated adjuvant radiofrequency ablation in areas of tumor receiving the lowest dose. The finite element method was used to solve Penne’s bioheat transport equation, coupled with the Arrhenius thermal cell-death model to determine 3D thermal ablation zones. Tumor and unablated tumor absorbed-dose metrics (average dose, D50, D70, D90, V100) following ablation were compared, where D70 is the minimum dose to 70% of tumor and V100 is the fractional tumor volume receiving more than 100 Gy.

**Results:**

Compared to radioembolization alone, ^90^Y radioembolization with adjuvant ablation was associated with predicted increases in all tumor dose metrics evaluated. The mean average absorbed dose increased by 11.2 ± 6.9 Gy. Increases in D50, D70, and D90 were 11.0 ± 6.9 Gy, 13.3 ± 10.9 Gy, and 11.8 ± 10.8 Gy, respectively. The mean increase in V100 was 7.2 ± 4.2%. All changes were statistically significant (*P* < 0.01). A negative correlation between pre-ablation tumor volume and D50, average dose, and V100 was identified (*ρ* < − 0.5, *P* < 0.05) suggesting that adjuvant radiofrequency ablation may be less beneficial to patients with large tumor burdens.

**Conclusions:**

This study has demonstrated that adjuvant ^90^Y PET/CT-guided radiofrequency ablation may improve tumor absorbed-dose metrics. These data may justify a prospective clinical trial to further evaluate this hybrid approach.

## Background

Tumor targeting in yttrium-90 (^90^Y) radioembolization differs from other radionuclide therapies that are infused systemically and find their targets through high affinity to cellular receptors. Instead, the distribution of ^90^Y within a tumor depends strongly on the catheter position during infusion, downstream fluid dynamics, and arterial perfusion in both tumor and uninvolved liver [1, 2]. Due to the mechanical nature of ^90^Y microsphere trapping, dose non-uniformities within tumor have been demonstrated through histological analyses [3–6] and ^90^Y PET/CT imaging [7–10]. While several metrics have been used to predict response following radioembolization [11], average tumor absorbed dose (*D*_avg_) is the most common metric. However, non-uniform deposition of ^90^Y may result in a sub-therapeutic dose to portions of the tumor, which has been shown to correlate with poor response even if *D*_avg_ is favorable [9].

Like many liver-directed therapies, ^90^Y radioembolization is commonly classified as a palliative treatment due to the relatively poor prognosis of patients with liver cancer. However, this does not suggest that there is no utility in treatment optimization. Response following locoregional hepatic therapy has been shown to correlate with improved patient survival, prompting the use of multimodality therapies [12, 13] to improve tumor response. One such example of multimodality therapy is combined trans-arterial chemoembolization (TACE) and radiofrequency ablation (RFA) for the treatment of hepatocellular carcinoma (HCC) [12]. Since the widespread use of post-radioembolization ^90^Y PET/CT, there has been an interest in utilizing this imaging data to improve patient therapy. While multimodality treatment utilizing ^90^Y radioembolization has not been widely studied, several authors have attempted the use of ^90^Y PET/CT to provide multistage patient-specific ^90^Y treatments with positive outcomes [14, 15].

Image-guided percutaneous ablation is the standard minimally invasive treatment for eligible patients with small (<3 cm) liver tumors [16]. However, the efficacy of ablation decreases with increasing tumor size. For patients with HCC, RFA generally results in poor outcomes for tumors greater than 5 cm in size, where alternative treatments such as ^90^Y radioembolization are commonly employed [17]. While combined RFA and TACE has demonstrated improved response in treating larger tumors [12], the utility of combining percutaneous ablation with ^90^Y radioembolization has not yet been evaluated. 3D ^90^Y PET/CT-based dosimetry and dose–response thresholds [18, 19] may be used in principle as a guide for percutaneous ablation of under-treated regions of tumor, potentially improving therapy.

In this work, ^90^Y PET/CT has been used as a guide for simulated modeling of adjuvant RFA. Improvement in tumor absorbed-dose metrics in the unablated tumor has been calculated to determine if this hybrid technique warrants further evaluation in a prospective clinical trial.

## Methods

The study was carried out in compliance with the Declaration of Helsinki and has been approved by the institutional review board at each participating site. Written consent was obtained from patients at site A (University of Tennessee IRB #3502), while a waiver of informed consent was obtained for data collected at site B (Wright State University IRB #SC6291). Patients treated with ^90^Y radioembolization using resin [site A, SIR-Spheres®, SIRTex Medical Ltd, North Sydney, Australia] or glass [site B, Therasphere® BTG, London, UK] microspheres for primary or secondary liver cancer and who received post-treatment ^90^Y PET/CT imaging were reviewed. Fourteen patients (age 49–80 years) met the inclusion criteria of tumor D70 < 100 Gy [9] for resin microspheres (D70 < 150 Gy for glass) with visualized areas of decreased microsphere uptake >2 cm in size identified on ^90^Y PET/CT, where D70 is the minimum absorbed dose (Gy) to 70% of the tumor volume. These patients received radioembolization for treatment of hepatocellular carcinoma (HCC, *n* = 10), intrahepatic cholangiocarcinoma (*n* = 2), or liver dominant metastatic disease (*n* = 2). The median model for end-stage liver disease score was 8, with 10 Child-Pugh class A patients and 4 class B patients. Additional data are available in Table 1.

**Table 1:** Patient demographic and ^90^Y radioembolization treatment data

Patient	Disease	Tumor volume (cm^3^)	Treatment device	Infused ^90^Y activity (GBq)
1	HCC	124.1	Resin	1.30
2	HCC	270.4	Resin	1.39
2	HCC	241.3	Resin	1.39
3	HCC	276.4	Resin	1.67
4	Cholangio	100.7	Resin	0.62
5	HCC	374.6	Resin	1.24
6	Cholangio	44.4	Resin	0.92
7	Endometrial	203.2	Resin	1.14
8	HCC	220.9	Resin	1.01
9	HCC	213.4	Resin	1.83
10	HCC	42.5	Resin	1.54
11	Breast	60.2	Resin	1.17
12	HCC	104.7	Glass	2.51
13	HCC	323.4	Glass	4.13
14	HCC	258.2	Glass	3.23

### Dosimetry

Patients treated at site A (resin microspheres) were scanned on a Siemens Biograph mCT Flow [Siemens Healthcare, Knoxville, TN] with a bed speed of 0.2 mm/s and reconstructed with 3D OSEM, 2i21s, time-of-flight, a 400 × 400 matrix (2.0 mm voxels), and no filter. Patients treated at site B (glass microspheres) were scanned on a GE Discovery 600 STE [GE Healthcare, Little Chalfont, UK] using two bed positions, 20 min per position, 3D OSEM, 2i24s, a 192 × 192 matrix (2.6 mm voxels), and no filter. ^90^Y imaging performance of these PET/CT scanners at activity concentrations common in radioembolization with resin microspheres has been evaluated previously [20]. All patients received post-radioembolization ^90^Y PET/CT imaging on the day of treatment.

Under the supervision of a dual-board-certified nuclear medicine radiologist, tumors were contoured in three dimensions on ^90^Y PET/CT referencing appropriate pre-treatment hepatic protocol CT or MRI in patients with HCC or ^18^FDG PET/CT images in patients with metastatic disease. Contours were drawn using Osirix 7.5 [Pixmeo, Bernex, CH], exported to Matlab 2014b [Mathworks, Natick, MA], and converted to a 3D mask with the same pixel size as ^90^Y PET/CT data. For each tumor, ^90^Y PET/CT-based 3D dosimetry was performed using the local deposition method [21] and dose–volume histograms were generated. The D50, D70, D90, maximum and average tumor dose (*D*_avg_), and V100 were calculated for each tumor since these metrics were previously validated as prognostic indices for radioembolization [9], where D50 and D90 are the minimum doses to 50 and 90% of tumor volume and V100 is the percentage of tumor volume receiving more than 100 Gy.

### Radiofrequency ablation simulation

Because RFA is the most common treatment in the USA for small tumor ablation in situ [16], it was selected as the simulated ablation modality in this study. While in vivo and ex vivo RF temperature profiles and ablation zones have been published previously, we elected to use biophysical modeling which allowed for flexible calculation of ablation zones for varied ablation times, electrode angles, positions, and tumor tissue characteristics (HCC or metastatic disease) which could be defined in a 3D voxel space matching that of ^90^Y PET/CT. This model was compared with data previously reported from similar models [22] and in vivo measurements [23] to establish its accuracy under a fixed set of conditions.

**Fig. 1:**
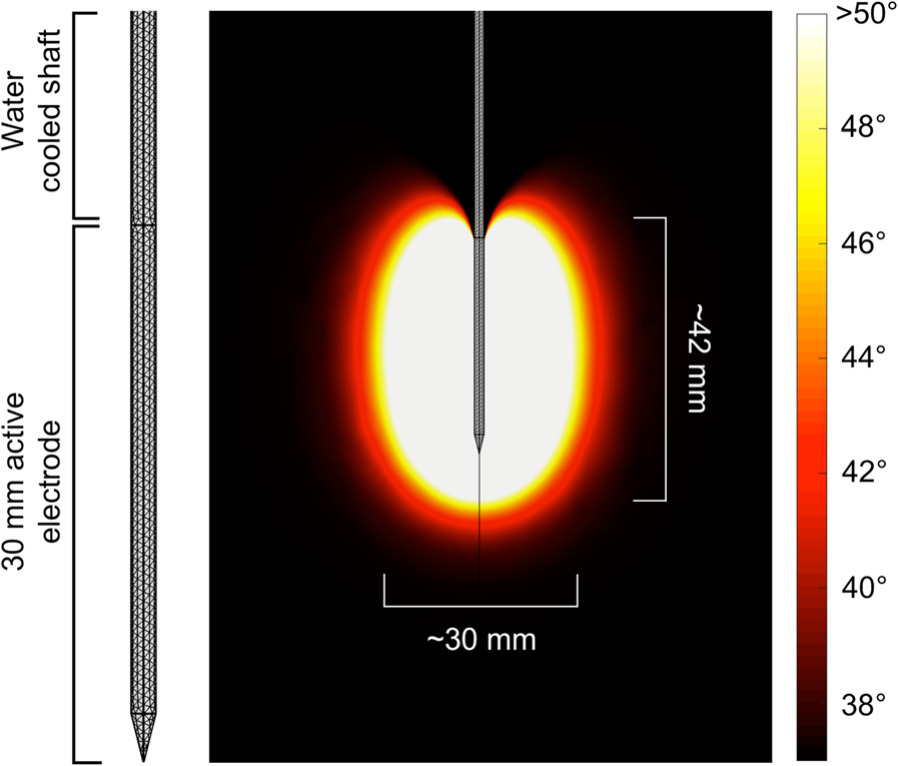
Model of actively cooled RF ablation electrode with tetrahedral mesh applied for FEM-based solution of the Penne’s bioheat transport equation (*left*). Simulated heating around electrode in HCC (Table 2) with RF ablation cell killing boundary (Ω = 6.9) axial and lateral range indicated (*right*)

The model consisted of a water-cooled 17-gauge straight RF electrode with a 30-mm active tip. The electrode model was placed inside a 20 × 20 × 20 cm block of liver tissue, with electrothermal properties discussed later in this section. A 3D tetrahedral mesh was applied to the model for finite element method (FEM) analysis of the heat transport problem. The maximum mesh element size was restricted to 8 mm in tissue, 1 mm in the insulating portion of the electrode, and 0.25 mm in the active electrode tip. The electrode model and tetrahedral mesh are shown in Fig. 1. The FEM was used to solve Penne’s bioheat transport equation [24], which consists of two partial differential equations:

1 $$ \nabla \left(\sigma \nabla V\right)=0 $$

2 $$ \rho c\frac{\partial T}{\partial t}=\nabla \left(k\nabla T\right)+{w}_{\mathrm{b}}{c}_{\mathrm{b}}\left({T}_{\mathrm{a}}-T\right)+{q}_{\mathrm{m}} $$

where ∇ is the Laplace operator, *σ* is the electrical conductivity (S/m), *V* is the electric potential (volts), *ρ* is tissue material density (Kg/m^3^), *c* is tissue specific heat (J/Kg/°C), *k* is the electrical conductivity of tissue (W/m/°C), *w*_b_ is the perfusion rate of blood per unit volume of tissue (m^3^/m^3^/s), and *c*_b_ is the specific heat of blood (J/Kg/°C). *T*_a_ is the temperature of arterial blood, held constant at 37 °C, and *T* is the temperature as a function of time, *t*. The initial conditions for all geometries at all nodes (tissue and electrode) were set to 37 °C, with liver boundary conditions maintained at 37 °C throughout the simulation. The potential (*V*) was modulated using a simulated feedback circuit to maintain the temperature at the hottest tissue node to <105 °C. The perfusion rate of blood per unit volume in tissue (*w*_b_) was varied during simulation to account for vascular coagulation [25]. The first-order kinetic Arrhenius model [25] was employed to estimate fractional vascular coagulation as a function of time and temperature during ablation, as described in Eq. 3.

3 $$ {C}_f=1-{e}^{-{\displaystyle {\int}_{t=0}^tA{e}^{\frac{-Ea}{RT(t)}}dt}} $$

*C*_f_ is the fraction of capillary flow that has been occluded, and *R* is the ideal gas constant (8.314 J/mol/°K). A is the Arrhenius pre-exponential factor, a measure of molecular collision frequency (1.98e106 1/s), and *E*_*a*_ is the activation energy, the amount of energy required to transform molecules from their original state to a damaged state (6.67e5 J/mol). *T*(*t*) is the temperature as a function of time. Following a previous investigation [25], *W*_b_ was linearly decreased with increasing *C*_f_ (Eq. 4) to simulate decreasing blood perfusion as RFA creates localized coagulation.

4 $$ {w}_{\mathrm{b}}={w}_{\mathrm{b},\mathrm{n}\mathrm{c}}\left(1-{C}_f\right) $$

In Eq. 4, *w*_b,nc_ is the perfusion rate of blood in the absence of any heat-induced vascular coagulation. The Penne’s heat transport equation [24] and similar approximations have been previously used for modeling of radiofrequency ablation in tissue, with several examples [22, 26] reviewing additional relevant details.

The RFA electrode model, tetrahedral mesh, and FEM analysis of the Penne’s bioheat equation were simulated as described using COMSOL (FEMLAB) 5.0 [COMSOL, Stockholm, Sweden]. Electrode angle, depth, ablation time, and tissue parameters were pre-defined through a Matlab interface with COMSOL for each location simulated. After each simulation, continuous temperature data as a function of time was re-binned in Matlab into a 3D spatial map with voxel sizes matching radioembolization data from quantitative ^90^Y PET/CT. Calculations were performed on a 6th-generation Intel Core-i7 system with 32 Gb of memory.

### Tissue parameters

Tissue parameters (*σ*, *ρ*, *c*, *k*, *c*_b_, *w*_b,nc_) were obtained from the literature for normal liver, cirrhotic liver, and HCC (Table 2). Tissue parameters for normal liver were used for the four patients treated for metastatic disease or cholangiocarcinoma (Table 1) since no data specific to these tumor types were available. Data for HCC were used for simulation in the remaining patients. For reference, data for cirrhotic liver, with characteristically decreased *w*_b,nc_, is also shown in Table 2.

**Table 2:** Tissue parameters used in RF ablation simulation

	*σ* (S/m)^a^	*ρ* (Kg/m^3^)^b^	*c* (J/Kg/°C)^c^	*k* (W/m/°C)^c^	*w*_*b,nc*_ (m^3^/m^3^/s), [(mL/min/100 mL)]	*c*_*b*_ (J/Kg/°C)^c^
Normal liver	0.260 ± 0.062	1060	3540 ± 118	0.52 ± 0.03	0.0180 ± 0.0057 [108 ± 34.0]^d^	3617 ± 301
Cirrhotic liver	0.260 ± 0.062	1040	3540 ± 118	0.52 ± 0.03	0.0115 ± 0.0050 [69.0 ± 30.0]^d^	3617 ± 301
Tumor (HCC)	0.504 ± 0.191	1060	3540 ± 118	0.52 ± 0.03	0.0155 ± 0.015 [92.8 ± 88.6]^e^	3617 ± 301

^a^Data from Haemmerich et al. [35]

^b^Data from International Commission of Radiation Units and Measures [36]

^c^Data from Hasgall et al. [37]

^d^Data from Schutt and Haemmerich [22]

^e^Data from Sahani et al. [38]

### ^90^Y PET/CT-Guided RFA

The tumor destruction boundary corresponding to each ablation location was calculated using the Arrhenius model for cell killing from FEM calculated time-dependent temperature (Eq. 5).

5 $$ - \ln (SF)=\varOmega (t)={\displaystyle {\int}_{t=0}^tA{e}^{\frac{-Ea}{RT(t)}}dt} $$

When applied to the prediction of thermal cell death, the Arrhenius pre-exponential factor (*A*) is 2.984e80 1/s, the activation energy (*E*_a_) is 5.06e5 J/mol, and SF is the surviving fraction of cells. An Ω value of 6.9 was selected, corresponding to a SF of 0.001 or 99.9% cell killing within the ablation zone.

Under the supervision of a board-certified interventional radiologist, up to four electrode locations were selected in each patient, with ablation time up to 600 s per location. Electrode locations were selected using realistic percutaneous access paths, as would be performed using conventional clinical techniques [16] under CT or CT fluoroscopy guidance, with the acquired ^90^Y PET/CT as a guide. Time and position were varied to limit the ablation zone to areas of tumor receiving <100 Gy when possible and, in cases where the ablation zone was close to the liver capsule or gallbladder, to spare these tissues. The ablation zone boundary was maintained at least 1 cm from nearby gastrointestinal tissue.

### Dosimetry with and without RFA

Following calculation of 3D ablation zones for each location, the Ω = 6.9 threshold was used to create a 3D mask of the absolute ablation boundary. This mask was subtracted from the pre-defined tumor mask, leaving unablated tumor, treated only with ^90^Y radioembolization. Dose–volume histograms (DVH), D50, D70, D90, *D*_avg_, and V100 were recomputed using the same 3D dosimetric dataset (less ablated tissue) to compare ^90^Y radioembolization with adjuvant PET/CT-guided ablation to radioembolization alone in this patient cohort.

### Statistical analysis

The Kolmogorov–Smirnov test was used to assess the assumption of normality for all datasets evaluated. Differences in tumor dose metrics between ^90^Y radioembolization and ^90^Y radioembolization with PET/CT-guided ablation were evaluated using a paired-sample *T* test. The potential correlation between both tumor size and number of ablation sites on post ^90^Y PET/CT-guided ablation dose metrics was assessed using the Spearman’s correlation statistic. Linear regression was used to explore this relationship if Spearman’s statistic suggested a correlation (|*ρ*| > 0.5). A *P* value <0.05 was considered statistically significant through all analysis.

## Results

Thirty-three RFA simulations were performed for the adjuvant simulated therapy of 15 tumors. The mean computation time per simulation was 7.8 min. Simulated ^90^Y PET/CT-guided RFA resulted in a calculated decrease in active tumor volume when cell killing inside the Ω = 6.9 boundary was assumed. The mean pre- and post-ablation tumor volume was 197.2 ± 102.3 cm^3^ and 179.3 ± 98.7 cm^3^, respectively. The average percent decrease in tumor volume was 12.1 ± 7.9%. Spearman’s statistic showed a moderate positive correlation between the number of ablation sites and the absolute change in tumor volume (*ρ* = 0.59, *P* = 0.02). Ablation and volumetric data for each tumor is shown in Table 3. Lack of consistency in the volumetric effect of ablation is secondary to varying overlap of ablation zones or ablation zones not fully contained within tumor boundaries.

**Table 3:** Summary of RF ablation locations and tumor volume change

Patient	Number of ablation sites	Ablation time (s)	Pre-ablation tumor volume (cm^3^)	Post-ablation tumor volume (cm^3^)	Percentage volume change
1	1	600	124.1	112.9	9.9
2^a^	3	600/600/600	270.4	239.7	12.8
2^a^	2	600/600	241.3	217.0	11.2
3	2	600/420	276.4	253.9	8.9
4	2	600/600	100.7	78.1	28.9
5	2	600/360	374.6	352.9	6.1
6	1	600	44.4	32.5	36.6
7	2	600/600	203.2	177.7	14.4
8	2	220/220	220.9	212.2	4.1
9	3	600/360/360	213.4	192.7	10.7
10	1	600	42.5	29.9	42.1
11	2	260/260	60.2	54.1	11.3
12	4	600/600/600/600	104.7	82.4	27.1
13	4	600/600/600/600	323.4	299.1	8.1
14	2	600/600	258.2	240.1	7.5

^a^Patient 2 had two large independent foci in different liver segments, which were treated independently in this analysis

### Tumor dose

**Fig. 2:**
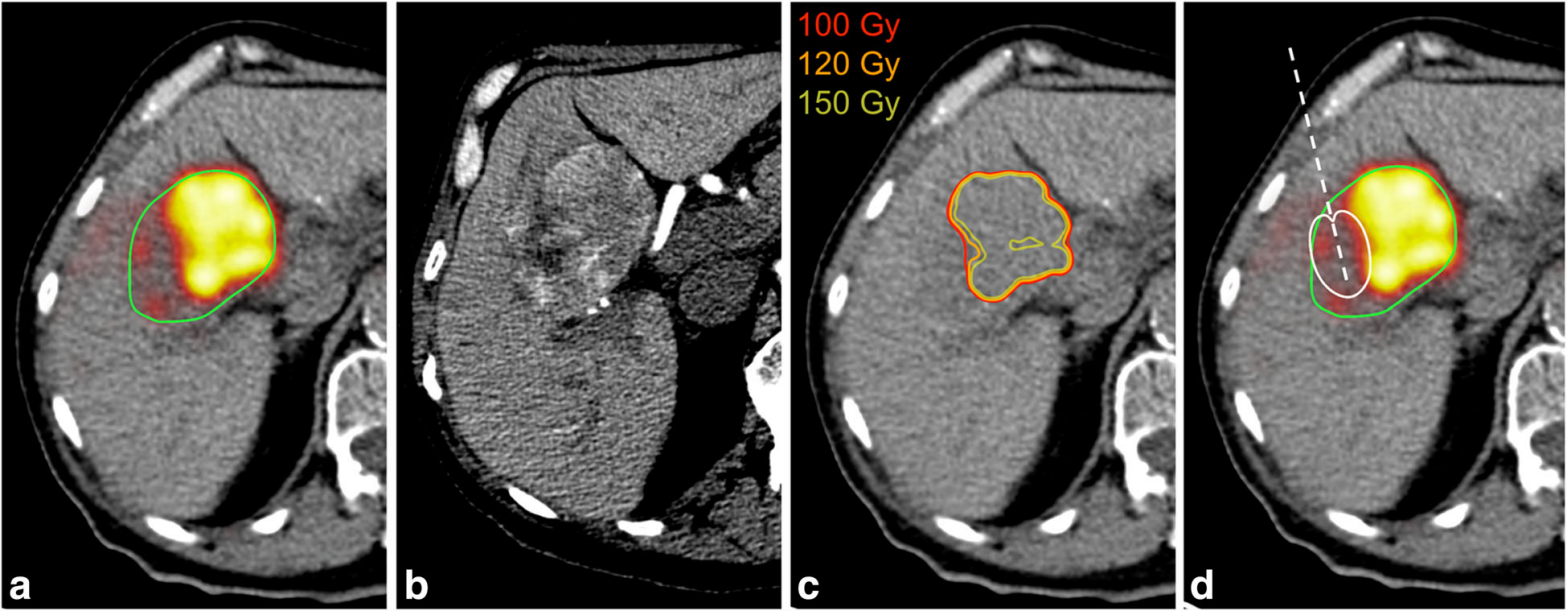
^90^Y PET/CT-guided treatment plan for patient 1 (Table 1). **a** Post-radioembolization ^90^Y PET/CT with tumor contour (*green*). **b** Pre-treatment hepatic protocol CT. **c** 100, 120, and 150 Gy isodose curves computed from post-radioembolization ^90^Y PET/CT using the local deposition method. **d** Electrode placement (*dashed white line*) and RF ablation cell killing boundary (*solid white line*, Ω = 6.9)

**Fig. 3:**
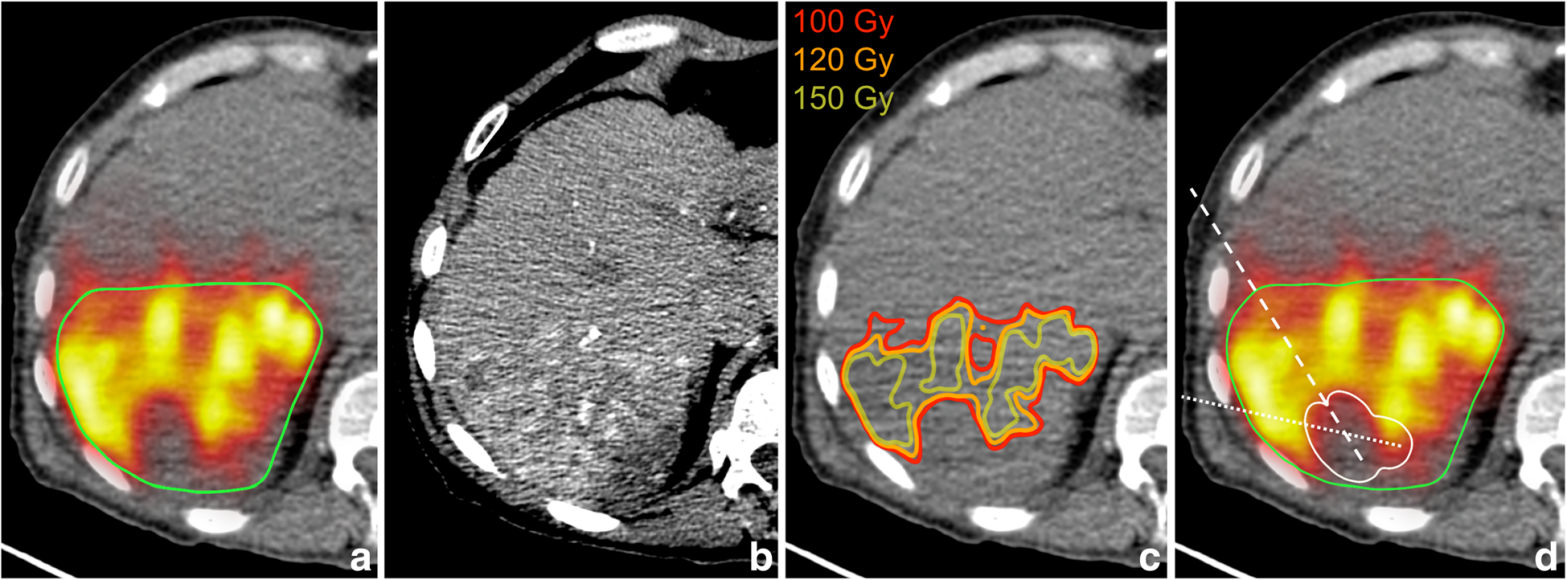
^90^Y PET/CT-guided treatment plan for Patient 5 (Table 1). **a** Post-radioembolization ^90^Y PET/CT with tumor contour (*green*). **b** Pre-treatment hepatic protocol CT. **c** 100-, 120-, and 150-Gy isodose curves computed from post-radioembolization ^90^Y PET/CT using the local deposition method. **d** Electrode placement in-plane (*dashed white line*), electrode placed 10 mm inferior to displayed slice (*dotted white line*) and RF ablation cell killing boundary (*solid white line*, Ω = 6.9)

**Fig. 4:**
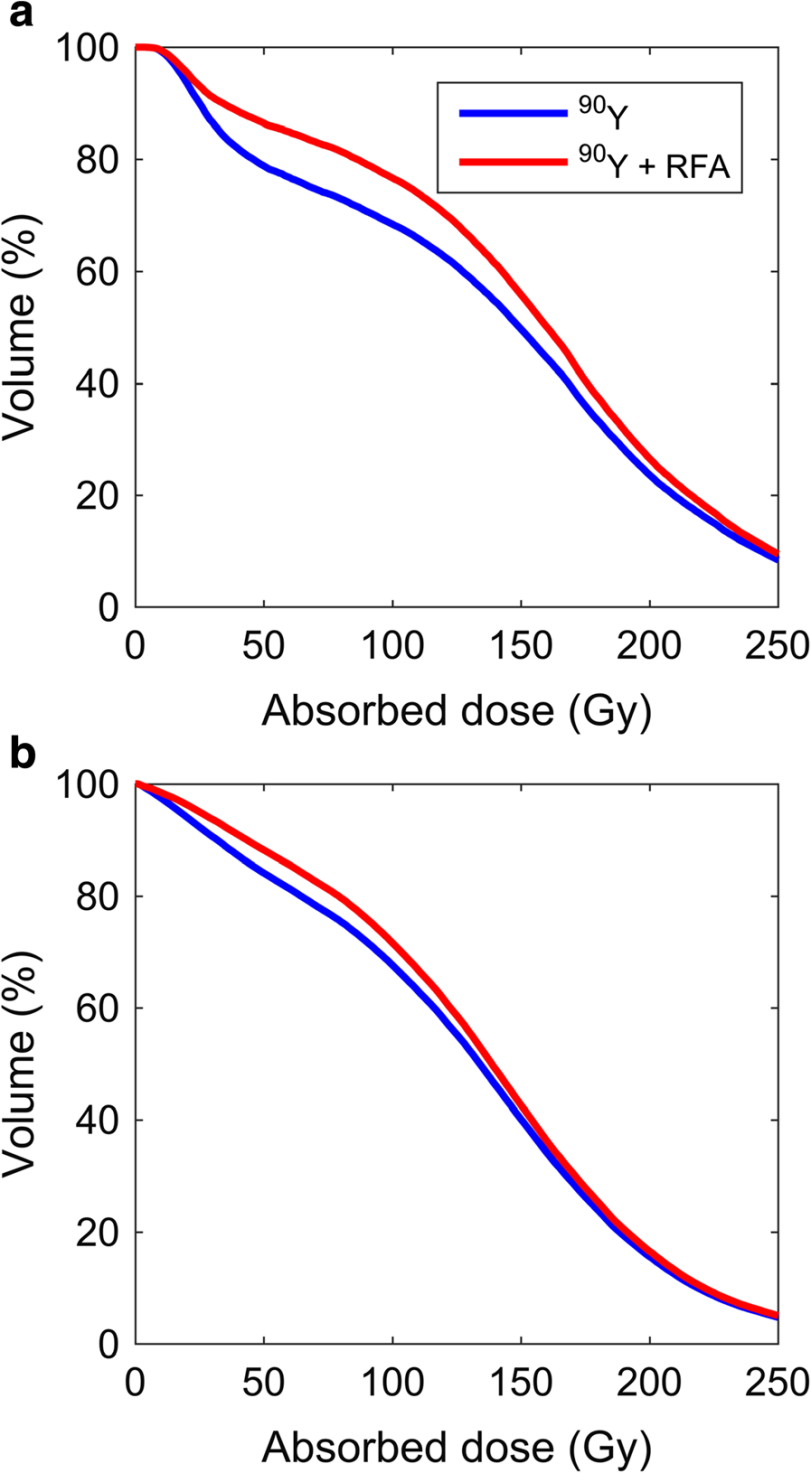
Dose–volume histograms for ^90^Y radioembolization alone and ^90^Y radioembolization with ^90^Y PET/CT-guided ablation. **a** Patient 1 (Table 1), pre-treatment tumor volume = 124.1 cm^3^. **b** Patient 5 (Table 1), pre-treatment tumor volume = 374.6 cm^3^

^90^Y PET/CT, particularly when converted to isodose contour plots at the 100-Gy level (Figs. 2c and 3c) or when the lower window level was set to 100 Gy, provided a clear guide for ablation. Examples of electrode placement for targeting of under-treated regions in two tumors spanning the full range of cohort tumor volume (patients 1 and 5, Table 1) are shown in Figs. 2 and 3. Adjuvant simulated ablation had the effect of modifying the shape of the DVH, reducing the percentage of tumor volume receiving lower absorbed doses in all cases. However, the effect was more pronounced for smaller tumors than larger tumors, as illustrated in Fig. 4a, b for patients 1 and 5, respectively.

In addition to affecting DVH shape, simulated ^90^Y PET/CT-guided RFA resulted in statistically significant increases in the *D*_avg_, D50, D70, D90, and V100 compared to ^90^Y radioembolization alone. The mean absolute increase in *D*_avg_ was 11.2 ± 6.9 Gy (*P* < 0.001). Mean increases in D50, D70, and D90 were 11.0 ± 6.9 Gy (*P* < 0.001), 13.3 ± 10.9 Gy (*P* < 0.001), and 11.8 ± 10.8 Gy (*P* < 0.01), respectively. The mean increase in V100 was 7.2 ± 4.2% (*P* < 0.001). Additional calculated dosimetric details comparing radioembolization alone and radioembolization with simulated RFA are given in Table 4. The limited volume of tumor that can be affected by RFA is reflected in the difference in the percent change in D50, D70, and D90. For example, D50 was increased by an average of 11.2%, D70 by 18.1%, and D90 by 43.8%. Since D90 is the minimum dose to 90% of the tumor volume, it shows the most drastic difference since adjuvant RFA targets a relatively small fraction of tumor volume receiving the lowest dose.

**Table 4:** A comparison of mean (*μ*), standard deviation (*σ*), and range for dose metrics evaluated with and without adjuvant ^90^Y PET/CT-guided percutaneous ablation

		^90^Y	^90^Y + RF ablation	Absolute change (Δ)	Percentage change (%)
*D*_avg_ (Gy)	*μ* ± σ	122.3 ± 39.5	133.5 ± 41.9	11.2 ± 6.9*	9.7 ± 6.7
	Range	67.3 − 210.2	72.1 − 231.6	2.2 − 21.9	2.0 − 23.4
*D*_max_ (Gy)	*μ* ± σ	523.2 ± 326.9	523.2 ± 326.9	0	0
	Range	223.1 − 1474.9	223.1 − 1474.9		
D50 (Gy)	*μ* ± σ	112.8 ± 43.3	123.8 ± 44.9	11.0 ± 6.9*	11.2 ± 8.5
	Range	48.5 − 201.8	52.9 − 225.3	2.5 − 23.6	2.4 − 26.7
D70 (Gy)	*μ* ± σ	77.9 ± 27.4	91.2 ± 32.4	13.3 ± 10.9*	18.1 ± 14.3
	Range	32.6 − 113.9	36.0 − 148.9	2.7 − 36.2	3.0 − 54.2
D90 (Gy)	*μ* ± σ	37.8 ± 18.1	49.7 ± 17.1	11.8 ± 10.8^†^	43.8 ± 55.5
	Range	14.1 − 72.9	19.4 − 74.4	1.4 − 43.7	1.9 − 216.6
V100 (%)	*μ* ± σ	52.8 ± 19.0	60.0 ± 18.2	7.2 ± 4.2*	16.9 ± 15.7
	Range	18.9 − 74.3	21.0 − 81.6	2.1 − 17.9	4.4 − 64.2

*P < 0.001; ^†^ *P* < 0.01

### Correlation with tumor volume

**Fig. 5:**
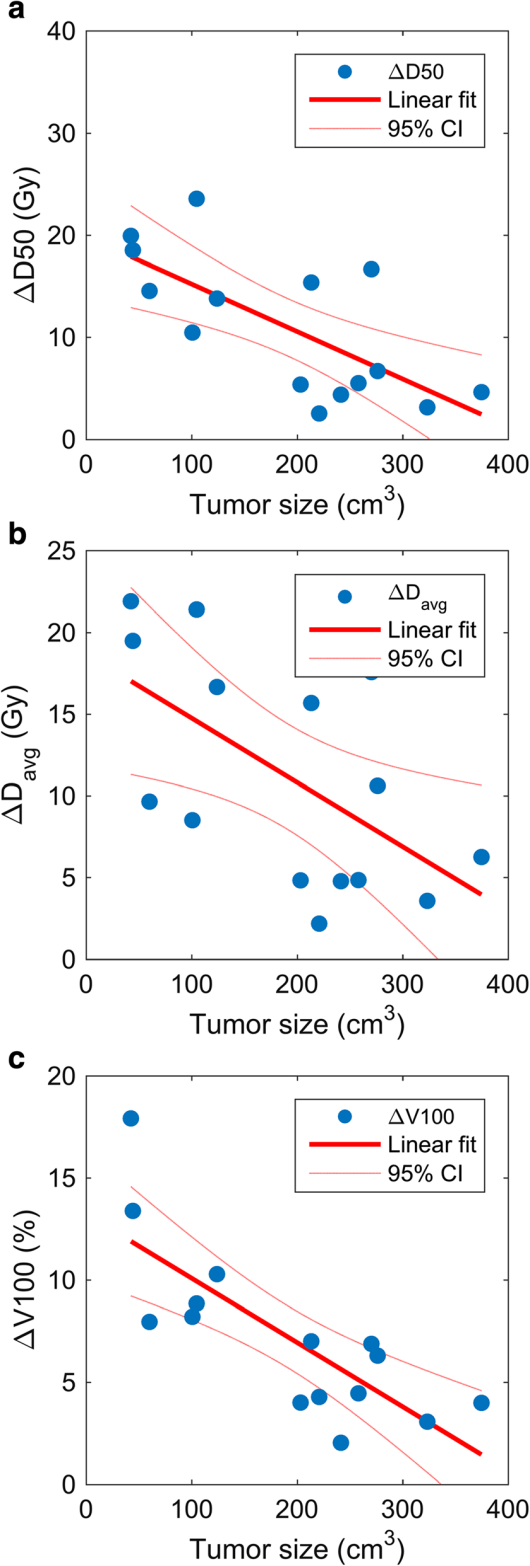
Linear regression for dose metrics vs tumor volume. **a** ∆D50 vs tumor volume (Slope_fit_ = −0.046 Gy/cm^3^, *R* ^2^ = 0.505, *P* = 0.003). **b** ∆*D*_avg_ vs tumor volume (Slope_fit_ = −0.04 Gy/cm^3^, *R* ^2^ = 0.354, *P* = 0.02). **c** ∆V100 vs tumor volume (Slope_fit_ = −0.034%/cm^3^, *R* ^2^ = 0.615, *P* = 5.4e−4)

Spearman’s statistic indicated a correlation between pre-ablation tumor volume and the absolute change in four tumor dose metrics (∆D50, ∆D70, ∆*D*_avg_, ∆V100) with adjuvant RFA. ∆D50 and ∆D70 exhibited a moderate (*ρ* = −0.64, *P* = 0.01) and weak (*ρ* = −0.34, *P* = 0.21) negative correlation with increasing tumor volume, respectively. No correlation between ∆D90 and tumor volume was observed (*ρ* = 0.14, *P* = 0.62), likely secondary to the sensitivity of D90 to the 10% of tumor volume receiving the lowest dose, which was effected by adjuvant ablation regardless of tumor size. ∆*D*_avg_ exhibited a moderate negative correlation (*ρ* = −0.54, *P* = 0.04) and ∆V100 showed a strong negative correlation (*ρ* = −0.8, *P* = 5.0e−4) with increasing tumor volume. Linear regression analysis for the dose metrics showing moderate or better correlation (|*ρ*| > 0.5) is summarized in Fig. 5. The *P* values in Fig. 5 confirm rejection of the null hypothesis when the linear fit is compared to a constant model. These analyses suggest that the effect of adjuvant RFA on ∆D50, ∆D70, ∆*D*_avg_, and ∆V100 is decreased with increasing tumor volume. On the other hand, ∆D90 remains favorable independent of the tumor size.

## Discussion

The distribution of ^90^Y radioembolization within the tumor can only be partially controlled by the treating physician, i.e., by careful microcatheter placement and through modification of downstream fluid dynamics. It is logical, therefore, to combine ^90^Y radioembolization with an adjuvant treatment modality that can be more precisely targeted, such as percutaneous ablation. Compared to RFA alone, there is substantial evidence that multimodality treatments such as combined TACE-RFA can result in improved survival in patients with intermediate size HCC lesions [12, 27]. While there are obvious similarities between TACE–RFA and combined radioembolization–RFA, it is the differences that warrant discussion within the context of the findings in this manuscript.

The primary utility of ^90^Y PET/CT is that it allows for proactive planning of alternative or adjuvant therapies immediately following radioembolization [14]. While not fully established, published absorbed-dose thresholds [18, 19] and quantitative ^90^Y PET/CT can be used to plan secondary therapies, potentially including the percutaneous ablation of areas of tumor receiving low absorbed dose. By the same token, areas of tumor receiving high absorbed doses can be spared superfluous secondary intervention. Unlike combined TACE–RFA, strict quantification of the change in absorbed dose, a physical quantity related to tumor response, can be predicted prior to adjuvant ablation following radioembolization—as has been done in this work. To this end, this study has demonstrated statistically significant improvements in both unablated tumor absorbed-dose metrics and DVH shape after simulated ablation following ^90^Y treatment in a 14-patient cohort.

The primary endpoint of this effort was successful in elucidating that improvement in unablated tumor dose metrics following adjuvant ablation appear possible; however, the clinical significance of this is unknown. In addition, neither the technical feasibility nor the complication rate associated with these two therapies in tandem has been evaluated. Because percutaneous ablation is highly targeted and spares normal liver tissue, however, it is likely that complication rates will be low. Radioembolization–RFA can be contrasted in theory to patients who received both external beam radiation therapy and ^90^Y radioembolization—a tandem treatment with a notable increase in hepatic toxicity [28].

The relationship between improved tumor absorbed-dose metrics and improved response has been demonstrated in ^90^Y radioembolization [9, 18]. However, there is sufficient variability in the dose–response thresholds reported in the literature that a prediction of improved treatment efficacy in the patient cohort analyzed in this manuscript cannot be made. The effect of combined radioembolization and percutaneous ablation on tumor response must, therefore, be evaluated as part of a future clinical trial. Such a trial focusing on outcomes would also allow exploration into situations where the tumor as a whole was grossly under-treated, but without substantial inhomogeneity. While adjuvant ablation in the case of homogeneous tumor dose would not result in a substantial change in dose metrics, outcomes may still be affected due to the concomitant biological effects of both treatment modalities.

One limitation of this study is that the only percutaneous ablation modality simulated was RFA. While RFA was selected due to its wide use in hepatic tumor ablation [16], radioembolization combined with other ablation modalities may be associated with different results. For example, the mean difference in pre- and post-RFA tumor volume among all patients was 18.9 cm^3^ (Table 3). This relatively small volume is not only due to lower ablation times used to target only under-treated tumor in some patients but also due to the relatively small RFA ablation zone size. In addition, the correlation identified between tumor volume and absolute change in several tumor absorbed-dose metrics (∆D50, ∆D70, ∆*D*_avg_, ∆V100) can be at least partially attributed to RFA ablation zone size. Alternatives such as microwave ablation are associated with larger ablation volumes may result in further improvement in unablated tumor absorbed-dose metrics if used following radioembolization.

A second limitation is that electrothermal tissue characteristics and in particular, *w*_b,nc_, can vary substantially and have a marked impact on calculated and actual RF ablation zone size [22]. To this end, it is likely that the model employed in this work, utilizing *w*_b,nc_ for normal liver and HCC (Table 2), resulted in an underestimation of the ablation zone size. One clinically well-known phenomenon in the thermal ablation of small HCC tumors in patients with cirrhosis is the “oven effect” [29] in which the decreased blood perfusion rate (*w*_b,nc_) in cirrhotic liver (Table 2) results in an increased ablation zone size. Since blood perfusion carries heat away from the electrode, it ultimately limits the ablation zone size regardless of the modality of thermal ablation used. However, in ^90^Y PET/CT-guided RFA, patients receive radioembolization with glass or resin microspheres prior to adjuvant ablation. The embolic nature of the microspheres, resin greater than glass, may decrease tumor blood perfusion and, therefore, could increase ablation zone size beyond that simulated in this work. The oven effect may be contributory to increased ablation zone size in RFA-TACE [12]; however, the effects following ^90^Y radioembolization cannot be determined at this pre-clinical stage.

Finally, this study has not considered the effects of respiratory motion on the ^90^Y PET/CT acquisition, tumor boundary delineation, calculated dose metrics, or the potential effect of respiratory motion on clinical electrode placement. Several studies [30, 31] have described changes in tumor volume and SUV in ^18^FDG PET/CT when respiratory gating was used. However, investigation into the feasibility of respiratory gating in ^90^Y PET/CT has been limited to a few examples [32, 33]. The usefulness of ^90^Y PET respiratory gating may be limited by the small branching ratio of positron emission in ^90^Y [34] and the clinical difficulty of increasing an already long acquisition time. Respiratory motion undoubtedly had an effect on the tumor dose metrics reported for each patient in this manuscript; however, the extent of which could not be quantified. As this effort proceeds into clinical investigation, more work into the potential use of amplitude based gating in ^90^Y PET [32] may be warranted and, at the very least, physicians must be aware of the potential effects of motion on ^90^Y PET data when planning adjuvant therapy.

Although this work precedes a clinical trial, the timing of radioembolization and subsequent ablation will be of concern and warrants brief discussion. ^90^Y radioembolization is a permanent implant, delivering 97.5% of its absorbed dose to tissue in the first 2 weeks after infusion. However, because there is no biological removal of ^90^Y, the absorbed dose is committed immediately after the microspheres are infused. In theory, the timing, therefore, between ^90^Y treatment and ablation is not critical so long as ablation is performed before structural tumor changes occur creating deviation with the ablation guidance model, i.e., the post-treatment ^90^Y PET/CT. One additional concern is whether heating of microspheres in situ could result in the release of free ^90^Y—a complication that must be investigated with in vitro experiments and patient bioassay before and during a future clinical trial. However, even if systemic release is found to be a potential issue, waiting two or more weeks before adjuvant ablation will allow nearly all the infused radioactivity to decay, minimizing safety concerns.

## Conclusions

This study has demonstrated that adjuvant ^90^Y PET/CT-guided ablation may improve tumor absorbed-dose metrics. These data may justify a prospective clinical trial to further evaluate this hybrid approach.

## References

[1] Kennedy AS, Kleinstreuer C, Basciano CA, Dezarn WA (2010). Computer modeling of yttrium-90-microsphere transport in the hepatic arterial tree to improve clinical outcomes. Int J Radiat Oncol Biol Phys.

[2] Basciano CA, Kleinstreuer C (2011). Computational fluid dynamics modeling of 90 Y microspheres in human hepatic tumors. J Nucl Med Radiat Ther.

[3] Campbell AM, Bailey IH, Burton MA (2000). Analysis of the distribution of intra-arterial microspheres in human liver following hepatic yttrium-90 microsphere therapy. Phys Med Biol.

[4] Campbell AM, Bailey IH, Burton MA (2001). Tumour dosimetry in human liver following hepatic yttrium-90 microsphere therapy. Phys Med Biol.

[5] Kennedy AS, Nutting CC, Coldwell DD, Gaiser JJ, Drachenberg CC (2004). Pathologic response and microdosimetry of 90Y microspheres in man: review of four explanted whole livers. Int J Radiat Oncol Biol Phys.

[6] Högberg J, Rizell M, Hultborn R, Svensson J, Henrikson O, Mölne J (2014). Heterogeneity of microsphere distribution in resected liver and tumour tissue following selective intrahepatic radiotherapy. EJNMMI Research.

[7] D’Arienzo M, Filippi L, Chiaramida P, Chiacchiararelli L, Cianni R, Salvatori R (2013). Absorbed dose to lesion and clinical outcome after liver radioembolization with (90)Y microspheres: a case report of PET-based dosimetry. Ann Nucl Med.

[8] Fourkal E, Veltchev I, Lin M, Koren S, Meyer J, Doss M (2013). 3D inpatient dose reconstruction from the PET-CT imaging of 90Y microspheres for metastatic cancer to the liver: Feasibility study. Med Phys.

[9] Kao YH, Steinberg JD, Tay YS, Lim GK, Yan J, Townsend DW (2013). Post-radioembolization yttrium-90 PET/CT—part 2: dose–response and tumor predictive dosimetry for resin microspheres. EJNMMI Research.

[10] Lhommel RR, van Elmbt LL, Goffette PP, Van den Eynde MM, Jamar FF, Pauwels SS (2010). Feasibility of 90Y TOF PET-based dosimetry in liver metastasis therapy using SIR-Spheres. Eur J Nucl Med Mol Imaging.

[11] Walrand S, Lhommel R, Goffette P, Van den Eynde M, Pauwels S, Jamar F (2012). Hemoglobin level significantly impacts the tumor cell survival fraction in humans after internal radiotherapy. EJNMMI Research.

[12] Carmi L, Georgiades C (2010). Combination percutaneous and intraarterial therapy for the treatment of hepatocellular carcinoma: a review. Semin Intervent Radiol.

[13] Newell PH, Wu Y, Hoen H, Uppal R, Thiesing JT, Sasadeusz K (2015). Multimodal treatment of unresectable hepatocellular carcinoma to achieve complete response results in improved survival. HPB (Oxford).

[14] Bourgeois AC, Chang TT, Bradley YC, Acuff SN, Pasciak AS (2014). Intra-procedural 90Y PET/CT for treatment optimization of 90Y radioembolization. J Vasc Interv Radiol.

[15] Chang TT, Bourgeois AC, Balius AM, Pasciak AS (2013). Treatment modification of yttrium-90 radioembolization based on quantitative positron emission tomography/CT imaging. J Vasc Interv Radiol.

[16] Gervais DA, Goldberg SN, Brown DB, Soulen MC, Millward SF, Rajan DK (2009). Society of Interventional Radiology position statement on percutaneous radiofrequency ablation for the treatment of liver tumors. J Vasc Interv Radiol.

[17] Livraghi T, Goldberg SN, Lazzaroni S, Meloni F, Ierace T, Solbiati L (2000). Hepatocellular carcinoma: radio-frequency ablation of medium and large lesions. Radiology.

[18] Strigari L, Sciuto R, Rea S, Carpanese L, Pizzi G, Soriani A (2010). Efficacy and toxicity related to treatment of hepatocellular carcinoma with 90Y-SIR spheres: radiobiologic considerations. J Nucl Med.

[19] van den Hoven AF, Rosenbaum CENM, Elias SG, de Jong HWAM, Koopman M, Verkooijen HM (2016). Insights into the dose–response relationship of radioembolization with resin 90Y-microspheres: a prospective cohort study in patients with colorectal cancer liver metastases. J Nucl Med.

[20] Willowson KP, Tapner M, Investigator Team QUEST, Bailey DL (2015). A multicentre comparison of quantitative (90)Y PET/CT for dosimetric purposes after radioembolization with resin microspheres: the QUEST Phantom Study. Eur J Nucl Med Mol Imaging.

[21] Pasciak AS, Bourgeois AC, Bradley YC (2014). A comparison of techniques for 90Y PET/CT image-based dosimetry following radioembolization with resin microspheres. Front Oncol.

[22] Schutt DJ, Haemmerich D (2008). Effects of variation in perfusion rates and of perfusion models in computational models of radio frequency tumor ablation. Med Phys.

[23] Goldberg SN, Ahmed M, Gazelle GS, Kruskal JB, Huertas JC, Halpern EF (2001). Radio-frequency thermal ablation with NaCl solution injection: effect of electrical conductivity on tissue heating and coagulation-phantom and porcine liver study. Radiology.

[24] Pennes HH (1998). Analysis of tissue and arterial blood temperatures in the resting human forearm. J Appl Physiol.

[25] He X, McGee S, Coad JE, Schmidlin F, Iaizzo PA, Swanlund DJ (2009). Investigation of the thermal and tissue injury behaviour in microwave thermal therapy using a porcine kidney model. Int J Hypertherm.

[26] Zorbas G, Samaras T (2014). Simulation of radiofrequency ablation in real human anatomy. Int J Hypertherm.

[27] Peng Z-W, Zhang YJ, Chen MS, Xu L, Liang HH, Lin XJ (2013). Radiofrequency ablation with or without transcatheter arterial chemoembolization in the treatment of hepatocellular carcinoma: a prospective randomized trial. J Clin Oncol.

[28] Lam MG, Abdelmaksoud MH, Chang DT, Eclov NC, Chung MP, Koong AC, Louie JD, Sze DY (2013). Safety of 90Y radioembolization in patients who have undergone previous external beam radiation therapy. Int J Radiat Oncol Biol Phys.

[29] Livraghi T, Goldberg SN, Lazzaroni S, Meloni F, Solbiati L, Gazelle GS (1999). Small Hepatocellular carcinoma: treatment with radio-frequency ablation versus ethanol injection. Radiology.

[30] Lupi AA, Zaroccolo MM, Salgarello MM, Malfatti VV, Zanco PP (2009). The effect of 18F-FDG-PET/CT respiratory gating on detected metabolic activity in lung lesions. Ann Nucl Med.

[31] Werner MK, Parker JA, Kolodny GM, English JR, Palmer MR (2009). Respiratory gating enhances imaging of pulmonary nodules and measurement of tracer uptake in FDG PET/CT. AJR Am J Roentgenol.

[32] Pasciak AS, Bourgeois AC, McKinney JM, Chang TT, Osborne DR, Acuff SN (2014). Radioembolization and the dynamic role of (90)Y PET/CT. Front Oncol.

[33] Mamawan MD, Ong SC, Senupe JM (2013). Post-90Y radioembolization PET/CT scan with respiratory gating using time-of-flight reconstruction. J Nucl Med Technol.

[34] Attarwala AA, Molina-Duran F, Büsing KA, Schönberg SO, Bailey DL, Willowson K (2014). Quantitative and qualitative assessment of yttrium-90 PET/CT imaging. PLoS ONE.

[35] Haemmerich D, Schutt DJ, Wright AS, Webster JG, Mahvi DM (2009). Electrical conductivity measurement of excised human metastatic liver tumours before and after thermal ablation. Physiol Meas.

[36] ICRU. International Commission on Radiation Units and Measurements. Tissue Substitutes in Radiation Dosimetry and Measurement. ICRU Report 44. Bethesda: International Commission on Radiation Units and Measurements; 1989b.

[37] Hasgall PA, Di Gennaro F, Baumgartner C, Neufeld E, Gosselin MC, Payne D, Klingenböck A, Kuster N (2015). “IT’IS Database for thermal and electromagnetic parameters of biological tissues,” version 3.0, September 01st.

[38] Sahani DV, Holalkere NS, Mueller PR, Zhu AX (2007). Advanced hepatocellular carcinoma: CT perfusion of liver and tumor tissue—initial experience. Radiology.

